# Medical treatment for an isolated renal multilocular hydatid cyst in an elderly: a case report

**DOI:** 10.1186/s12882-020-02064-5

**Published:** 2020-10-08

**Authors:** Atie Moghtadaie, Seyed Amir Miratashi Yazdi, Minoo Mohraz, Hoda Asefi, Effat Razeghi

**Affiliations:** 1 Internal Medicine Department, Sina hospital, Tehran University of Medical Sciences, Tehran, Iran; 2 General Surgery Department, Sina hospital, Tehran University of Medical Sciences, Tehran, Iran; 3 Iranian Research Centre for HIV/AIDS (IRCHA), Department of infectious diseases, Tehran University of Medical Sciences, Tehran, Iran; 4 Sina hospital, Tehran University of Medical Sciences, Tehran, Iran; 5 Nephrology Research Center, Tehran University of Medical Sciences, Tehran, Iran; 6 Center of Excellence in Nephrology, Tehran University of Medical Sciences, Tehran, Iran

**Keywords:** Case report, Hydatid cyst, Medical treatment, Renal mass

## Abstract

**Background:**

Almost all cases of renal hydatid cysts need surgical intervention for treatment. We report a case of isolated renal hydatid cyst treated successfully only with medical therapy.

**Case presentation:**

This case is a 79-year-old veterinarian presented with right flank pain, hydatiduria and positive echinococcus granulosus serology. A 70*50 mm cyst with daughter cysts in mid-portion of right kidney on presentation was changed into a 60*40 mm cyst without daughter cysts at last follow-up. Due to patient’s refusal of surgery, our patient received medical treatment including praziquantel and albendazole. After completion of first round of treatment, recurrence occurred and the same treatment was repeated. At last, the cyst became inactive and calcified with negative serology and no clinical symptoms under medical treatment.

**Conclusion:**

The treatment of choice in renal hydatid cyst is surgery; although there are some reports about the efficacy of medical treatments for hydatid cysts but lower rates of recurrence and higher efficacy put surgery in a superior position compared to medical approaches. Our case showed relative success of medical treatment, despite the presence of a large multilocular renal involvement. Thus, medical therapy without surgery can be considered in very particular cases with isolated renal hydatid cysts.

## Background

Hydatid cysts are caused by echinococcus species. Echinococcus is a flat worm with three evolutionary stages during its life cycle. The structure of this cyst is usually composed of three components. A component is a “pericyst” which is produced by the inflammatory tissues of the host. The other two elements are “exocysts” and “endocysts” [1, 2]. Iran is an endemic region for hydatid disease. The prevalence of hydatid cysts in Iran has been reported to vary from 1.2 to 13.8% in different parts of the country [3].

Risk factors of development of hydatid cysts include direct contact with animals such as sheep and dogs, as well as geographic region and consumption of contaminated water and raw vegetables. This infection can affect different organs of the body. The most common involved parts of the body are liver (~ 50–70% of cases) and lung [4]. Involvements of spleen, brain and kidney are also reported in the literature. Isolated renal involvement is very rare and accounts for about 2% of the all cases [5].

Renal involvement in hydatid cysts can be symptomatic or asymptomatic. The diagnosis is based on history, imaging, pathological and serological findings. The enzyme-linked immunosorbent assay (ELISA) test has an 80% sensitivity at initial diagnosis for hydatid cyst [6]. Laboratory findings in this infection are non-specific and vary based on the involvement of different organs but leukocytosis, eosinophilia and hypogammaglobulinemia are expected in involvement of any organ of the body.

On imaging evaluations, a thin calcification rim can suggest hydatid cyst. In ultrasound examinations, the presence of daughter cysts can be a key for diagnosis of hydatid cyst but CT scans are more sensitive for detection of hydatid cysts [7].

Treatment of choice for renal hydatid cyst is surgical removal. Although medical therapy has been reported in treatment of a few cases but high rates of recurrence with medical therapy prompt the need for longer follow-up duration in patients [8]. Successful treatment of isolated renal hydatid cysts is rare and hereby we report our case as an example of treatment of this condition without surgery.

## Case presentation

A 79-year-old male retired veteranarian from Semnan province, living in Tehran, was referred to a non-academic private nephrology clinic due to slightly elevated creatinine levels and flank pain at 2012. He had hypertension and coronary artery stenting. He was under treatment with aspirin and metoprolol.

In 2005, he had presented with generalized itching and a skin lesion on his left leg, which was non-significant on biopsy and resolved without any special treatment. He was totally asymptomatic until 2012, when he was admitted at a private clinic hospital in Tehran, for intermittent right flank pain without urinary symptoms.

Ultrasonography revealed cysts in the kidneys. Four cysts with the largest diameter of < 3 cm and one cyst of 36*49 mm size with multiple septa in right kidney were visualizied. There were four cortical cysts in the left kidney with a maximum diameter of 28 mm. The patient had been discharged with the wrong diagnosis of bilateral simple cysts and was advised for continuation of follow-up.

After 3 years, the patient presented at our hospital for the first time with acute-onset severe right flank pain radiating to groin which lasted for 6 h. He also complained of nausea, vomiting, hematuria and passage of small, white, balloon-like, and grape-size structures in urine. Laboratory findings were listed in Table 1.

**Table 1:** Lab findings at intial presentation and last follw-up

Parameter	At initial presentation	At last follow-up
Hemoglobin	14.9 g/dL	14.6 g/dL
WBC	7 × 10^9^/L	6.3 × 10^9^/L
- Neutrophils	51%	49%
- Lymphocytes	26%	38%
- Eosinophils	15%	7%
CRP	Not detectable	Not detectable
Urea	30 mg/dL	24 mg/dL
Creatinine	1.5 mg/dL	1.2 mg/dL
AST	15 U/L	12 U/L
ALT	12 U/L	13 U/L
Antibodies specific for Echinoccus granulosis (IgG)	Positive	Negative
U/A	Blood: 1+ Bacteria: Few WBC: 5 RBC: 6	Blood: Negative Bacteria: Few WBC: 3 RBC: 1

*WBC* white blood cells, *CRP* C-reactive protein, *AST* aspartate aminotransferase, *ALT* alanine transaminase, *IgG* immunoglobulin G, *U/A* urine analysis

**Fig. 1:**
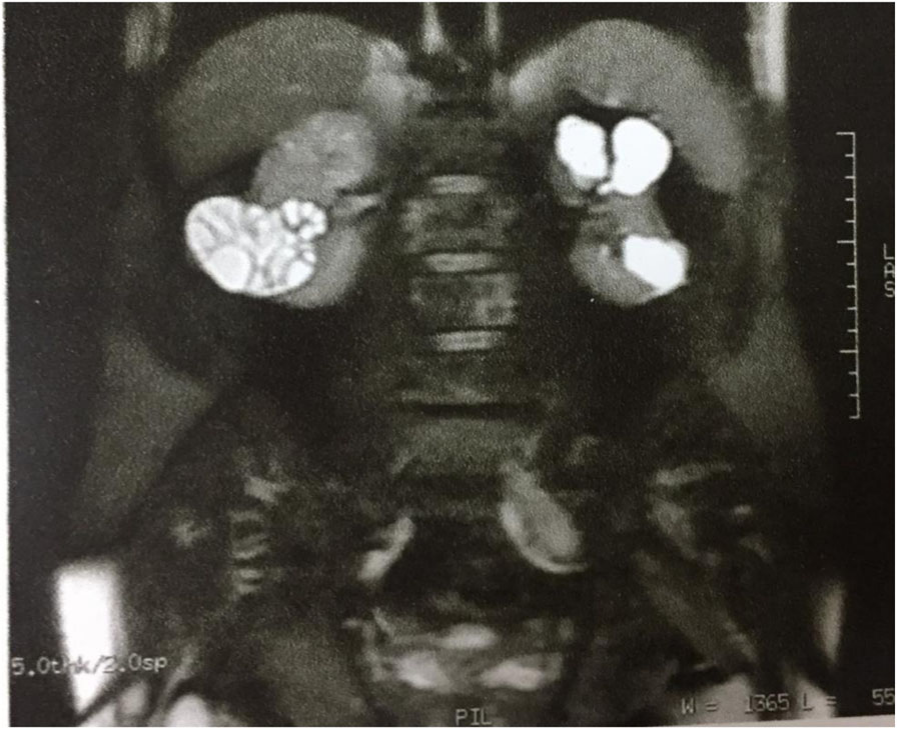
MRI image of the patient on initial admission at our center showing multilocular cyst at right kidney

**Fig. 2:**
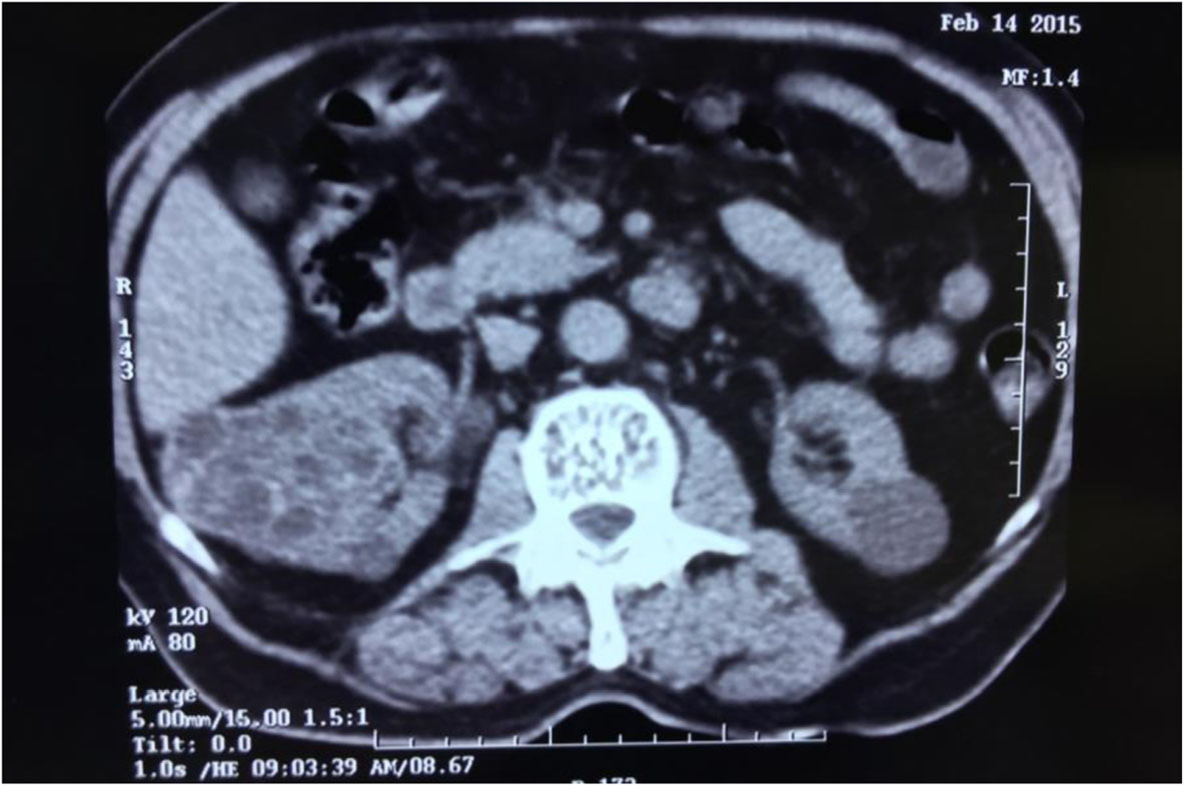
CT-scan of the patient on initial admission at our center showing multilocular cyst at right kidney

The kidneys were examined by ultrasonography and there were some cortical cysts in the left kidney with a maximal diameter of 33 mm and fine calcified septa in one cyst. There was also a solid mass containing small cystic regions with the size of 60*74 mm in lower pole of right kidney with external extension and no vascular flow on color Doppler ultrasonography. The bladder was reported to be normal. Since a complicated cyst was suspected, further investigations were performed. In CT scan, a 70*50 mm multiloculated cystic lesion with multiple non- enhancing internal septa resembeling walls of doughter cysts in mid portion of right kidney was reported (Figs. 1 and 2).

The pathological assessment of balloon-like structures in urine revealed that they were highly suggestive of hydatid cyst. Pathological evaluation showed a laminated cyst wall, partially lined up by a layer of germinal cells. In serological assessment, there were weakly positive results in two laboratory assays by ELISA with a five-month interval.

Due to the diagnosis of active hydatid cyst, surgery was recommended but the patient refused surgical treatment. The reason for refusal was the patients’ severe fear of undergoing surgery. Then praziquantel 800 mg three times a day and albendazole 400 mg three times a day were initiated for the patient. Follow-up was recommended.

Fourteen months later, while under treatment, he was presented with hydatiduria. Pathology again confirmed the diagnosis of hydatid cyst. With recurrence of hydatiduria, surgical treatment was offered to the patient again, but he refused and medical treatment was continued. Around 12 months later, the hydatid cyst serology turned negative and after 3 months, he was recommended to stop the medications. The concurrent complete blood count, liver function tests and urinalysis were normal.

**Fig. 3:**
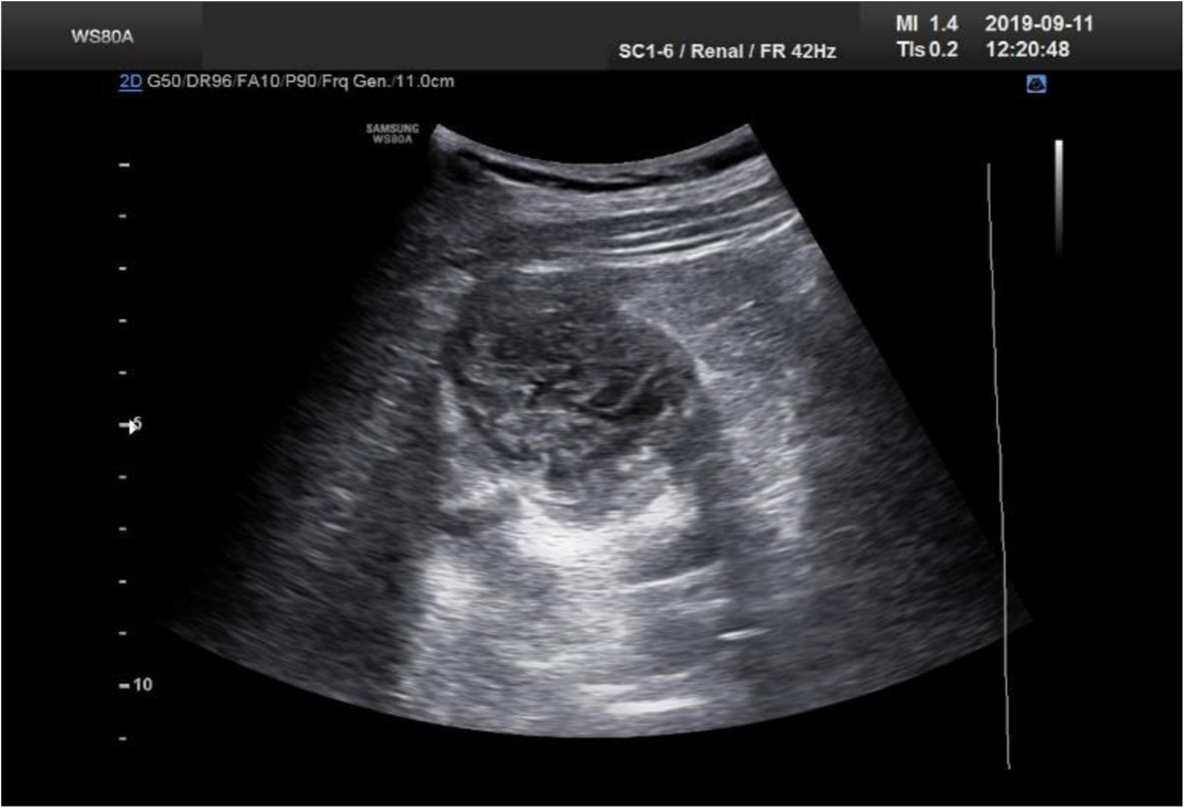
Ultrasonographic evaluation 3 years after discontinuation of medications showing disappearance of daughter cysts

Nine months after cessation of medical treatment, ultrasonography showed a hetero-echo exophytic mass in the right kidney with a size of 58*42 mm. There were cortical cysts in the left kidney in the middle-lower region with the maximum diameter of 26 mm. The bladder wall was thickened. In CT scan a 60*44*42 mm cystic lesion without obvious internal septa and no enhancement was seen. Calcification in the cyst was observed. Follow-up sonography at the last visit 3 years after discontinuation of medications, demonstrated a 60*40*45 solid-appearance lesion without daughter cyst in mid portion of the right kidney (Fig. 3). At last, no clinical symptoms were present and the size of lesion was under control. The lesion was turned into a CE4 lesion from an initial CE2 lesion (Table 2).

**Table 2:** Comparison of lesion degrees by different classifications

	2020	2015
Gharbi ultrasound classification	3	4
Morphology	2a	4
WHO classification	CE4	CE2

*WHO* World Health Organization

## Discussion and conclusion

Hydatid disease is a parasitic infection, which can be transmitted from animals to humans. The disease is prevalent all over the world and some areas such as Iran are considered endemic for this disease, as traditional animal husbandry is prevalent in Iran [9].

Other countries such as Greece, Australia, and Turkey [10] are involved with this disease. As mentioned before, various body organs may be invovled and different symptoms may develop. Symptoms range from fever and shivering to symptoms specifically related to involvement of each organ. Symptoms may initially be silent but as the cyst grows, the patient develops complications [11]. The cyst may become large enough to destroy the tissues of the host. Renal hydatid cyst is rare and accounts for 2% of all hydatid cyst cases [12].

Early diagnosis and treatment of the disease are the key steps in preventing complications. The patient may be asymptomatic for many years or present with flank pain, hematuria and hydatiduria. Sometimes it can manifest as a cortical cyst. According to Gharbi sonographic classification of hydatid cysts, type I is a simple cyst without internal architecture [13]. Thus, careful review of history, occupational exposure and physical examination of patient is necessary to proceed to further investigations, if indicated [14].

Patient first presented at 2012 with flank pain. Back then, he was diagnosed with simple cyst labelled as Bosniak class I renal cyst. If more careful history was taken, more advanced evaluations such as serology, repeat ultrasonography and CT scan could be ordered and the delay in diagnosis and consequent complications could be prevented. History of the patients can give the clinicians valuable clues for the diagnosis. As in our case, being a veterinarian increases the likelihood of presence of hydatid cysts. In patients with high-exposure backgrounds, additional evaluations could prompt the appropriate diagnosis [14].

If the cyst invades the urinary tract, it causes hydatiduria, which is a pathogenomonic finding for hydatid cyst. Hydatiduria occurs in 10–20% of cases, which is usually microscopic. Gross passage is relatively uncommon, although it has high diagnostic value [15]. Eosinophilia occurs in 50% of the cases, as in our case [16]. Serology in absolute kidney hydatosis is usually negative, however, our patient had two positive serological tests in the course of the disease.

Simple abdominal radiography reveals nonspecific findings which are mostly not useful for establishing the exact diagnosis. Ultrasound performed by an expert radiologist who can detect daughter cysts and other features of hydatid cyst can be useful but in majority of cases, these findings are missed on ultrasonography. CT-scan is the modality of choice for imaging in hydatid cyst which has high diagnostic yield as high as 98% specificity [17].

In renal hydatid cysts, parenchymal preservation surgeries such as cyst removal or partial nephrectomy are the main treatment options. Nephrectomy is also indicated in cases where the cyst is ruptured into the cavity and is complicated by a kidney infection ultimately leading to sequels in kidney [18]. However renal sparing procedures are the treatment of choice for most cases, As in our patient who refused this procedure. Histopathological examination of the nephrectomy specimens is the method of choice for confirmation of the diagnosis; but our case was diagnosed by a pathological assessment of hydatid cyst and serological assessments.

Ramteke et al. reported a 50-year-old female patient with 6-month history of lower abdominal pain presenting with hematuria. Hematuria as the presenting complaint was the same as our case but the difference is the location of pain. In this case abdominal pain was reported but in our case, flank pain was notable. She had right renal hydatid cyst on imaging which was treated with right nephrectomy and albendazole treatment regimen before and after operation [12]. Kumar et al. [18] reported a 37-year-old female patient with right iliac fossa pain. The patient had a shorter clinical course compared to our patient [18]. A case series of 11 patients with renal hydatid cyst was reported by Zargar et al. [19]. Among these patients, only two patients were older than 60 years of age. In addition, majority of cases were males and almost all cases were treated by surgery. The important point in that study is the age and gender distribution of patients. Although our patient was over 70 years of age, it is expected that this disease occurs in earlier stages of life. The superiority of male gender is reported in other studies. For instance, Huang et al. reported that 15 out of 19 patients with renal hydatid cysts were males [20]. It is speculated that this gender distribution can be attributed to higher occupational exposures in males.

Surgery is the treatment of choice in renal hydatid cyst. According to cyst size, location and staff expertise, it could be managed by total or radical nephrectomy. In some cases, conservative surgical excision and drainage are also recommended; although medical treatment is needed to prevent dishydatosis as well as spillage. The medical treatment usually includes albendazole before and after surgery [21].

In very rare studies, only medical therapy has been used to treat renal hydatid cysts- particularly in younger patients [22]. In the study by Soares et al. [22], a renal hydatid cyst with the size of 9*9*6.8 cm in a 14-year-old patient was treated with 4-week cycles of albendazole. The result was reduction of cyst size to 3.6*3.4*2.3 cm and seronegativity for hydatid disease. In our case, due to the patient’s refusal of the surgical treatment, we implemented the medical treatment and significant improvement in the size, volume, and clinical symptoms of the patient was achieved. Anyway, recurrence occurred once and treatment took longer. During the treatment, no adverse effects such as leukopenia, anaphylactic reaction, liver toxicity or alopecia were seen. Based on this case we reported, medical therapy alone can be an alternative for patients who refuse to undergo surgery. This treatment choice must be preserved for very limited cases. Using combination of albendazole and praziquantel in our case showed relatively favorable outcomes in controlling the disease without any significant adverse effects. Albendazole has been reported to be the most effective drug for inhibiting the cyst growth and praziquantel is used for killing worms in the gastrointestinal tract and treating protoscolices in larval stages [23]. Previous experiences with this combination in treating human hydatic cyst disease have proven effective in disseminated and non-operable cases [24]. For instance, Jamshidi et al. [25] reported disappearance of symptoms in 77.8% of patients and partial response in 22.2% of patients 18 months after receiving combination of albendazole and praziquantel. Radiologic evaluations also revealed significant response in 55.6% and partial response in 44.4% of patients.

According to the case we reported, medical therapy with the combination of albendazole and praziquantel can be used with comparable outcomes for patients who can not undergo surgery or refuse the surgical procedure. In these cases closer monitoring and follow-up must be conducted due to higher probability of recurrence of cyst.

## Data Availability

All the relevant data and materials of our patient are present in the manuscript but in case the original copy of the documents are needed, they are available to be delivered by the authors to the journal.
